# Effect of Local Heterogeneities on Single-Layer DNA-Directed Protein Lattices Through Non-Averaged Single-Molecule 3D Structure Determination

**DOI:** 10.21203/rs.3.rs-6095207/v1

**Published:** 2025-04-04

**Authors:** Gang (Gary) Ren, Jianfang Liu, Shih-Ting Wang, Meng Zhang, Zijian Hu, Hao Wu, Oleg Gang

## Abstract

Programmable and self-assembled two-dimensional (2D) protein lattices hold significant potential in synthetic biology, nanoscale catalysis, and biological devices. However, achieving high-order 2D lattices from three-dimensional (3D) nanoscale objects remains challenging due to structural heterogeneity caused by the flexibility and distortions of building blocks and their connectivity in a unit cell, leading to the formation of lattices with imperfections. This flexibility largely limits the analysis of key structural parameters at unit-cell resolutions due to the need to average 3D reconstructions in current methods. Here, we utilized advances in individual-particle cryo-electron tomography (IPET) to analyze the 3D structure of a designed 2D lattice formed by DNA-origami octahedral cages (unit-cell particles) encapsulating ferritin by determining the non-averaged 3D structure of each unit-cell particle. These protein-carrying DNA cages were analyzed at ferritin loading percentages of 100%, 70%, and 0%. Correlation analysis revealed that neither the ferritin loading percentage nor off-centralized placement in cages significantly affected lattice parameters, flexibility, or long-range order. Instead, the soft nature of DNA cages and interparticle linkages were the primary reasons for lattice imperfections. Structural improvements for enhancing lattice orders were evaluated through a series of molecular dynamics simulations. The developed cryo-EM 3D imaging reveals the molecular origin of heterogeneity of DNA-origami 2D lattices and highlights a path toward improved lattice designs.

## Introduction

Proteins, integral components of the molecular machinery in all living organisms, have evolved to fulfill distinct functional roles [1] through their specialized structures or by organizing themselves into complex 2D lattices [2,3], such as S-layers [3]. Inspired by nature, artificial 2D protein materials, including designed analogues [3,4] and de novo-designed arrays [5], have been developed for nanomedicine [6,7], nanoelectronics [8,9], and nanophotonics [10]. Beyond programmable and self-assembled 2D protein lattices marking a crucial step toward 3D crystals [4,5], the development of innovative 2D biomaterials, including designed analogues [3,6] and de novo-designed arrays [7], is highly sought after for applications in nanomedicine [8,9], nanoelectronics [10,11], and nanophotonics [12]. The functionality of these 2D materials often relies on the formation of highly ordered structures. However, synthesis frequently yields lattices with varying degrees of imperfections, necessitating structural investigation to refine the synthesis process and understand the causes of their origin.

A typical approach to determining the 3D structure of protein 2D lattices at high resolution is through crystallographic methods, such as X-ray and cryogenic electron crystallography [13-16]. In this technique, the lattice, embedded in vitrified ice to maintain its near-native state, is reconstructed into a 3D map through the combined analysis of electron microscopic images and diffraction patterns acquired at different tilt angles. Although near-atomic resolution can often be achieved from highly ordered lattices [17], obtaining an intermediate-resolution 3D map from a low-ordered lattice remains challenging.

Moreover, crystallographic methods do not provide information about the local heterogeneities and structural details that cause lattice imperfections.

Alternatively, real-space imaging can reveal the structure of a 2D lattice through cryogenic electron microscopy (cryo-EM) single-particle averaging (SPA) analysis [18]. While this approach has successfully achieved a high-resolution 3D structure of protein without relying on crystallography, it requires selecting the most homogeneous populations from a large pool of unit-cell particles and averaging them into one or a few static structures[19]. This selection process, focused on the most common and stable structure, obscures the details of individual unit-cell particles, which may represent unique or less common structures that often play crucial roles in inducing lattice imperfections.

Cryogenic electron tomography (cryo-ET) [20,21] is a promising method for determining the 3D structure of a single biological object by reconstructing 3D structures from images taken at a series of tilting angles, without relying on averaging, selection, or symmetric constraints (**Fig. 1a**). Cryo-ET has been used to investigate the non-averaged ultrastructure of single microscale objects, such as a bacterium or a cell slice [22]. However, several challenges complicate the non-averaged 3D structural determination of nanoscale objects [23], such as a protein particle, a DNA/RNA origami particle, or a unit-cell particle within a disordered lattice. These challenges include small physical dimensions, weak signals, low signal-to-noise ratio (SNR) resulting from radiation damage, the limited number of tilted images, overlapping features within a compact organization, and artifacts due to the missing wedge effect from restricted tilt angle ranges [24]. Common solutions involve sub-tomogram averaging by selecting and averaging of a homogeneous population of 3D maps into a single structure. Although this strategy reduces noise and missing-wedge artifact, key bottleneck remains in the use of an averaging approach, which cannot mitigate intrinsic errors present in the original low-resolution reconstruction, and thus unable to identify particles with unique conformations within a lattice that often contribute to defects in the lattice order.

We address these challenges by leveraging individual-particle cryo-electron tomography (IPET), a technique that enhances the resolutions of 3D reconstructions of individual macromolecular particles without the need for averaging. Over the past decade, we have advanced IPET capability for analyzing non-averaged 3D structures of small biological objects, such as single proteins or macromolecular complex particles. The developed methods include: i) a focused electron tomography reconstruction (FETR) algorithm, which reduces the effects of large-scale image distortion, ice- and lens-induced deformation, and tilting angle/axis readout errors[25]; ii) an image contrast enhancement technique [26]; iii) a low-tilt tomographic 3D reconstruction (LoTToR) algorithm to restore missing-wedge data caused by limited tilt angle range [27]; and iv) fully automated mechanical data acquisition software [28]. To date, IPET has achieved resolutions ranging from 2 to 10 nm [29-32], approaching the theoretical limits [33].

In this study, we utilized IPET to reveal the heterogeneity in 2D lattices and its origins by determining the non-averaged 3D structures of each unit-cell particle within the lattice, without relying on a pre-given initial model, particle selection, symmetry enforcement, or averaging with different unit-cell particles. The model of 2D lattice is assembled from protein-encapsulating octahedral DNA cages, which consist of 12 helix bundles (HBs), each contains 6 double-stranded DNA (dsDNA) helices with 84 base pairs [34,35](**Supp. Fig. 1**). Ferritin, a ~450 kDa protein composed of 24 subunits involved in iron storage [36], was engineered with single-stranded DNA (ssDNA), and encapsulated inside the DNA cages via 8 complementary linkers. Although 2D and 3D lattices have been successfully self-assembled from these designed particles, the resulting lattices might possess imperfections whose origin cannot be revealed by typically employed X-ray scattering methods. Our previous work has demonstrated highly programmable 2D and 3D lattices by decoupling ferritin's structural and functional nature from the DNA frameworks. Notably, our solution X-ray scattering results have initially indicated low lattice orders in 2D but with limited information on the casualty of flexibility and disorders.[37] Understating the local conformations of DNA origami in lattices is critical for revealing the origin of lattice imperfections and for guiding future engineering of DNA-based materials.

To uncover the structural heterogeneity of a planar lattice formed from 3D DNA cages, the non-averaged 3D structure identification of all elements within the lattice is required. First, we examined the hypothesis that the encapsulation of ferritin can affect DNA cages and, subsequently, cause lattice imperfections. For this purpose, we examined three types of 2D lattices assembled from octahedral DNA-cage particles encapsulating ferritin [2] at different loading levels, 100%, 70%, and 0%. Moreover, through a comprehensive analysis of the distributions of structural elements (spatial distribution of cages and their features, cage occupancy, inter-origami linkers, and ferritin positions), we found a correlation between individual structural dynamics and overall lattice disorder. Through molecular dynamics (MD) simulations [34]and simulated small-angle X-ray scattering (SAXS) spectra, we identified the unique regions that caused the lattice disorder.

## Results

#### Morphology of low-ordered 2D lattices

We first examined a 2D lattice composed of DNA cages encapsulating ferritin with nearly 100% loading (**Fig. 1a, Extended Data Fig. 1a**). Cryo-EM micrographs of the lattices embedded in vitrified ice revealed the overall morphology, identified as $P_{422}$ symmetry (**Fig. 1b, Extended Data Fig. 1b**). Fourier transforms of these images displayed the highest order of diffraction spots at (±3, 0) and (0, ±3), corresponding to ~18 nm (top-right corner in **Fig. 1c**, **Supplementary Data Fig. 1c**). Both primitive lattice vectors measure ~56 ± 2.5 nm (mean ± standard deviation, sd.), with an interfacial angle of ~90°. These unit-cell dimensions were consistent with the previous measurements obtained through SAXS [2]. The absence of diffraction spots at resolutions higher than 18 nm indicates the lattice heterogeneity and its origin can be revealed through analysis of all individual elements of the lattice.

We first investigated the impact of ferritin loading onto cages on a lattice order, motivated by the hypothesis that ferritin can perturb cage shape, leading to the distortions of inter-cage bonds. We analyzed two additional samples with reduced ferritin loading percentages: ~70% and 0% (**Extended**

**Data Fig. 1d,e,h,i**). Survey micrographs and Fourier transforms confirmed that the $P_{422}$-like symmetry was maintained across all lattices. The primitive lattice vectors measured ~56 ± 2.7 nm for 70% ferritin-loaded lattice and ~55 ± 2.8 nm for the lattice without ferritin, values consistent with the 100% ferritin-loaded lattice (**Extended Data Fig. 1f,g**). The highest diffraction spots were consistently observed at (±3, 0) and (0, ±3), reaffirming the reduced lattice order. Interestingly, the lattice without ferritin exhibited slightly more diffraction spots, while the lattice with 70% ferritin loading displayed slightly fewer. This suggests that ferritin loading influences the lattice intrinsic order but does not significantly affect long-range ordering.

**Non-averaged structure of an individual cage particle**

Zoomed-in images of the DNA cage particles forming 2D lattice revealed 8–12 rod-like densities, ~5–10 nm in diameter and ~20–40 nm in length, arranged in a quadrilateral pattern with intersecting diagonal rods at the center (**Fig. 1d**). The dimensions and characteristics of these rods are consistent with the designed 6-helical bundles (HBs) of double-stranded DNA (dsDNA), which self-assemble into an octahedral cage [2]. However, the structural symmetry of these cages is disrupted by curvatures and kinks along the rods, as well as missing connections at several distal ends (indicated by arrows in **Fig. 1d**).

Inside the cages, a ring-like particle with a diameter of ~10 nm (**Fig. 1b,d**), appears nearly at the cage center, consistent with the presence of encapsulated ferritin. The success in direct visualization of both the HBs and the encapsulated ferritin suggests the potential for 3D reconstruction of individual cage and cage-ferritin particles without ensemble averaging. The observed curvatures of the HBs, disconnected vertices, and off-center ferritin particles highlight the break in symmetry and the intrinsic structural variability inherent to the cage particles and, by extension, the lattice they form.

To capture the spatial arrangement of individual cages within a 2D lattice, we acquired a tilt series of images using cryo-ET (**Supp. Video. 1**) and reconstructed a 3D density map via ab initio 3D reconstruction of each individual unit-cell particle within the lattice at a resolution of ~3–9 nm (**Fig. 1e, Extended Data Fig. 2a,** and **Supplementary Table 1**).

To illustrate the detailed procedure of IPET 3D reconstruction of an individual unit-cell particle, the intermediate steps of 3D reconstruction are shown, with representative tilt projections demonstrated progressive signal enhancement of the cage structure as tilt-series alignment accuracy improved (**Fig. 1e-h,** and**Extended Data Fig. 2a**). Perpendicular cross-sectional views and central slices of the final 3D map revealed a quadrilateral-like structure with a central density (**Fig. 1f**). Six orthogonal views of the final 3D map showed an octahedral-like cage formed by 12 rod-like densities surrounding a central particle (**Fig. 1h**). Some vertices were fully connected by four rods, while others exhibited gaps, suggesting an asymmetric structure.

Notably, some rods displayed detailed structural features, such as fiber-like or dot-like arrangements of the helices (**Extended Data Fig. 2b,c**). Further analysis involved cropping and separately displaying two individual rods from the IPET 3D map. Each rod-like density showed six fiber-like densities, with each fiber measuring ~2 nm in diameter, consistent with a dsDNA helix within the 6-HB (**Extended Data Fig. 2d-p**). The visibility of individual dsDNA helices in the unit-cell particle suggests that the IPET 3D map achieved a resolution at ~1.6 to 2.1 nm. However, due to the complex and dense design of the 6-HB—composed of multiple dsDNA helical staples—the current resolution was insufficient to distinguish each dsDNA within the fiber-like densities to achieve a fully fitted 6-HB model. Nevertheless, the map was adequate for the rigid-body fitting of the 6-HB model into the rod-like densities in the IPET map (**Extended Data Fig. 2i,j and o.p**).

By fitting the structural models of each of the 12 6-HBs into the rod-like density fragments within the IPET map (**Fig. 1i**) and inserting the ferritin structure (PDB entry: 1IER [38]) into the core density, we generated an asymmetric structural model of the unit-cell particle. This model revealed that three quadrilateral-shaped 6-HBs were assembled nearly perpendicular to one another.

**Non-averaged 3D structures of 145 unit-cell particles**

Next, we obtained another 145 non-averaged 3D density maps of all 145 unit-cell particles within two representative lattices by repeating the above IPET 3D reconstruction protocol. The first five particles #1-5 were selected from a part of the huge lattice for testing the IPET capability (**Fig. 2a, Supp. Fig. 2)**, and the rest 140 particles #6-145 were reconstructed from a whole lattice (cages) within a come from another set of tomo2 (**Extended Data Fig. 3a**). Each map consisted of 12 rod-like densities arranged in an octahedral like cage with one or two core densities. Among these maps, 140 unit-cell particles contained one ferritin, 24 contained two ferritin particles, one contained three ferritin particles, and one contained none (**Extended Data Fig. 3a**). These results indicate a minimum ferritin occupancy of 118%, aligning with the ~35% ferritin overload during the experimental procedure [2]. Notably, some ferritin particles were observed on the exterior of the cages rather than inside, suggesting excess of unbound ferritin can be non-specifically hosted by lattice in a space between the cages.

By following the same rigid-body fitting process on each IPET 3D map, 140 cage-like fitting models were achieved (**Supp. Video. 1**), albeit without symmetry constraints. Upon aligning and superimposing all 140 models onto a standard octahedral model, the centers of the vertices, formed by the four nearby 6-HBs, were found to cluster around the vertices of the ideal octahedral model (**Extended Data Fig. 4a**).

To analyze the structural diversity of these 140 fitting models, the structural differences between each pair of models were calculated using root-mean-square deviations (RMSDs). The distribution of the RMSDs was then analyzed through hierarchical clustering analysis. The 1D dendrogram plot showed that the structures were nearly evenly distributed across clusters and sub-clusters, with similar subgroup sizes (**Extended Data Fig. 4b**), suggesting that the unit-cell particles experience thermal-like vibrations.

To investigate the inherent mechanism of the observed deviations, we first measured internal angles formed by adjacent HBs. The angles within each of the three quadrilaterals (one aligned with the lattice plane, shown in **Extended Data Fig. 4c** and two nearly perpendicular to it) revealed average angles of ~89.7° ± 4.9°, ~89.8° ± 5.4°, and ~89.8° ± 6.4° respectively. The internal angles, being close to 90°, suggest that all three quadrilaterals are nearly square in shape. However, the higher standard deviations of the internal angles in the two vertical quadrilaterals compared to the in-plane quadrilateral indicate greater flexibility in the vertical direction, Further analysis showed a strong correlation (R = 0.9) between the internal angles of the quadrilateral along the lattice plane and those perpendicular to it (**Extended Data Fig. 4d-g**).

The high correlation suggests that while the four in-plane HBs are relatively rigid in length and shape, they are connected via a mechanical linkage, where movement in one direction influences motion in the perpendicular direction. Additionally, dihedral angles analysis of the quadrilateral along the lattice plane revealed angles close to 180°, which were highly correlated with each other (**Extended Data Fig. 4h**), indicating that the quadrilateral may function as a Bennett linkage mechanism [30]. Analysis of the directions of the two off-plane vertices relative to the center of the in-plane "Bennett linkage" showed a peak population tilted ~5–10° away from the normal direction of the plane (**Extended Data Fig. 4i**), further suggesting the observation that the top and bottom vertices are tilting.

To further analyze the structural flexibility of the unit-cell particle, the particle size was measured by determining the vertex-to-vertex distances along two diagonal directions within the in-plane Bennett linkage (**Fig. 2f**). The histograms of these measurements showed a near-Gaussian distribution of the distances along both directions, indicating thermal vibrations.

Additionally, the size of the pores at each vertex, formed by the four distal ends of the 6HBs, was analyzed. The averaged pore size in the vertical direction was larger than in the horizontal direction (8.4 nm vs. 5.7 nm), suggesting that the top and bottom vertices exhibit greater flexibility compared to the in-plane vertices linked with vertices from neighboring octahedra (**Fig. 2g, Extended Data Fig. 5a,b**). This observation aligns with the higher standard deviations of internal angles measured in the vertical direction.

To evaluate the variability in particle volumes, we calculated the cage volume by modeling a polyhedron formed by the 24 vertices. The volume distribution was measured at ~30,150 ± 1,508 $nm^3$ (**Fig. 2h, Extended Data Fig. 5c,d**), which is ~7.2% larger than the designed octahedral structure and the volume obtained through the single-particle averaging (SPA) method (~28,138 $nm^3$). Notably, particles encapsulating two ferritin particles were slightly larger than those encapsulating one, increasing by ~2.8%, suggesting that ferritin loading is unlikely to cause significant distortion in the unit-cell particles.

Analysis of the central positions of encapsulated spherical ferritin within each unit-cell cage particle revealed that the ferritins were not precisely centered within the cages, showing an averaged off-center distance of 7.1 ± 5.1 nm (**Fig. 2i, Extended Data Fig. 5e,f**), despite being designed to occupy the exact center. This off-centering indicates that not all 8 inner linkers were successfully conjugated to the ferritin surface or that all these linkers were hybridized with DNA strands inside cages. The presence of particles containing two or more ferritin particles further confirms that free linkers remained available to bind additional ferritin particles.

These findings indicate that the unit-cell particles exhibit asymmetric structure, differing from the octahedral structures typically observed in conventional cryo-EM SPA analyses [39,40]. This discrepancy may arise from the enforcement of symmetry constraints during the initial or entire refinement process in SPA analysis.

### 3D structure of the entire low-ordered 2D lattice

To achieve a 3D density map of the entire lattice, the 140 high-resolution IPET 3D maps were superimposed into a low-resolution 3D map of the entire lattice (**Fig. 2b, Extended Data Fig. 3b, Supp. Video 1**). The low-resolution map was generated using IMOD software [41], while the positioning of the IPET map in its original unit-cell location was computed using Chimera software [42]. In the reconstructed map of the entire lattice **(Fig. 2c**), the IPET maps provide higher resolution details of the unit cells, while the IMOD map provides the precise positions and orientations between unit cells. Initial survey views revealed that the reconstructed lattice map exhibited near $P_{422}$ symmetry. Statistical analyses determined that unit-cell vector lengths to be ~54.7 ± 2.7 nm and ~56.1 ± 2.1 nm, with a peak interfacial angle of ~90.0° ± 3.8° (**Fig. 2e, Extended Data Fig. 6a-c**).

At the next step, we analyzed lattice distortion using the 3D density map we had obtained. The cage centers relative to the lattice fitting plane were measured and then plotted as displacement vectors along the fitted lattice plane (**Fig. 2d**). The distribution of the vectors showed that the unit-cell centers did not share a single plane, with some cages located above and others below the average lattice plane. The continuous changes in displacement created ripples on the lattice, resembling those observed in 2D materials, such as graphene [43]. Further analysis indicated that these ripples showed no correlation with the orientation or distortion of the unit-cell structures themselves, suggesting that the ripples are related to the thermally-induced lattice fluctuations in solution, which has been frozen in their momentary state in a cryogenic environment.

The unit-cell particles also exhibited rotation within the lattice plane and tilting against the plane (**Extended Data Fig. 7a**). Rotation was measured by the direction of two opposite vertices within the in-plane quadrilateral relative to its nearest lattice direction (**Supp. Fig. 3a**). The histogram revealed a wide distribution in the range of ±20° with a peak population of ~-2° (**Extended Data Fig. 7b**). The heat map showed the angle were evenly distributed across the lattice (**Extended Data Fig. 7c**), suggesting a degree of rotational freedom for the unit cells, likely due to flexible inter-vertex linkers.

The tilt angle was measured as the direction between the top and bottom vertices of the unit-cell particles relative to the normal direction of the lattice fitting plane (**Extended Data Fig. 7a**). The histogram showed that unit cells often tilt against the lattice plane, with a wide distribution from ~-4° to ~45° and a peak population around ~12° (**Extended Data Fig. 7b**). However, ~3.4% of the particles (~10 particles) exhibited significant tilting relative to the lattice plane, with angles ranging from ~30° to ~45°. The heat map showed an evenly distributed tilt angle across the lattice, including highly tilted unit cells (**Extended Data Fig. 7d**), further indicating rotational and tilting freedom due to flexible linkers.

To further analyze the freedom between adjacent unit cells, the nearest distances between two adjacent unit cells were measured, named linker distal-distal distance The histograms of the linker distal distances along the X- and Y-axis were similar, with values ranging from ~3 to ~18 nm and a peak population around ~9 nm (**Supp. Fig. 5d,e**). These distances are significantly shorter than those in the standard model, i.e. 17.2 nm (based on the distance of the 22-base ssDNA + 8-base-pair dsDNA + 22-base ssDNA, **Fig. 4a**). The shorter linker distance suggests that the ssDNA within the linker provides insufficient stiffness to maintain the intended separation between unit cells. The soft linkers allow additional freedom, enabling unit-cells rotation and tilting, which further disrupts lattice asymmetry and long-range order.

#### Correlation analysis of flexibilities between unit-cell particles and their formed lattice

Lattice distortions and short-range order can arise from various factors, including large-scale bending and ripples, local flexibilities of the unit-cell particles (cages), flexibilities of the linkers between cages, free rotation and tilting of the cages, and structural distortions induced by ferritin loading. To identify the primary causes of lattice distortion or disorder, we conducted a correlation analysis between lattice parameters and cage structural characteristics, including size and flexibility.

The lattice parameters, measured as the center-to-center distances between adjacent cages, exhibited weak correlations (R = 0.22–0.28) with cage sizes, defined as the diagonal vertex-to-vertex distances (**Supp. Fig. 4a-c**). This result suggests that distortions in the cage structure alone are not the primary drivers of lattice disorder. Similarly, the correlations between lattice parameters and linker properties—such as linker lengths or angles measured between nearest vertices of adjacent unit-cell particles—were also weak, with correlation coefficients ranging from R = 0.46–0.56 (**Supp. Fig. 5a-e**). These findings indicate that the flexibility of the linkers is not solely responsible for lattice disorder.

In contrast, a combined analysis of the cage and linker flexibilities revealed a significantly stronger correlation (R = 0.91–0.95) with lattice distortion (**Fig. 2j, Supp. Fig. 3c-e**). This combined measure was defined as the sum of three distances: the linker length between the two nearest vertices and the distances from these vertices to their respective cage centers. These results emphasize that the combined flexibilities of both the cages and their linkers are critical contributors to lattice distortion. These analyses highlight the synergistic role of cage structure and linker flexibilities in driving lattice disorder, offering deeper insights into the structural dynamics underlying lattice distortions.

#### Effects of ferritin loading on the lattice

To assess whether ferritin loading affects lattice parameters and order—an important consideration for the biological applications of crystal sponges—we conducted experiments on two new lattice types: one with 70% ferritin loading and another with no ferritin loading (**Fig. 3, Extended Data Fig. 8,9**). For the 70% ferritin-loaded lattice, 3D reconstruction was performed on 70 unit-cell particles. Of these, 55 encapsulated one ferritin particle, 3 encapsulated two ferritin particles, and 12 contained no ferritin (**Fig. 3a-d, Extended Data Fig. 8, Supp. Video 2**). For the 0% ferritin-loaded lattice, 3D reconstruction was performed on 122 unit-cell particles, none of which encapsulated ferritin (**Fig. 3e-h, Extended Data Fig. 9, Supp. Video 3**).

Statistical analyses of the lattice parameters and unit-cell sizes for the 70% ferritin-loaded lattice revealed no significant differences compared to the fully ferritin-loaded lattice. Key metrics such as lattice lengths and angles, unit-cell particle sizes, pore sizes, and the off-center distances of encapsulated ferritin showed similar distributions (**Extended Data Fig. 5g-i**). Additionally, analyses of in-plane rotations and tilting angles of unit-cell particles were consistent with those of the fully ferritin-loaded lattice (**Extended Data Fig. 6d,e, and 7e-g**). These results indicate that the process of ferritin loading does not significantly impact lattice stability or order.

Further analysis of the lattice without ferritin loading supported this conclusion. Distributions of lattice lengths and angles, unit-cell particle sizes, volumes, pore sizes, as well as rotation and tilt angles closely matched those of the 100% and 70% ferritin-loaded lattices (**Extended Data Fig. 5j,k, 6f,g, and 7h-j**). These findings confirm that lattice flexibility is not induced by variations in ferritin loading levels but instead depends on the intrinsic properties of the unit-cell structure and the linker design.

Based on these observations, we propose that enhancing the stability of the two most flexible vertices within the unit-cell and reinforcing the linkers between particles could improve lattice stiffness. Such improvements may result in a more stable and higher-order lattice structure, enhancing its potential for biological applications.

**Lattice dynamics revealed by MD simulations**

To investigate the underlying causes of lattice heterogeneity, MD simulations were conducted on the unit-cell particle and the lattice they form under various conditions, including different temperatures, lattice sizes, linker models, and ferritin loading levels (**Supp. Fig. 6a**). Due to the computational complexity posed by the large number of atoms (over 370,000 per unit-cell particle), all-atom MD simulations were not feasible. Instead, a coarse-grained DNA model at the nucleotide level, oxDNA, along with its visualization tool, oxView, was employed for the simulations [44,45].

Unit-cell simulations: Simulations of a single unit-cell particle—a DNA origami cage without encapsulated ferritin—were conducted for ~0.27 μs at 277 K (experimental temperature) and 300 K (room temperature, commonly used for oxDNA simulations). RMSD analysis indicated structural stabilization after ~0.04 μs (**Supp. Fig. 6b**). Analyses of pore size and tilt angles revealed no significant differences in distributions between the two temperatures (**Supp. Fig. 6c-f**), suggesting that temperatures in the range of 277–300 K do not significantly influence the flexibility of the unit-cell particle or the lattice it forms.

Lattice simulations: Simulations of 5 × 5 lattices with both parallel and crossing linkers were performed at 277 K for up to ~3 μs, under both empty (**Supp. Video 4**) and ferritin-loaded conditions (**Fig. 4g,f, Supp. Fig. 6g-j**). RMSD analysis showed that unit-cell particles near the lattice boundary exhibited higher flexibility in their orientations compared to those at the center. To minimize boundary effects, only the central unit-cell particle was used for statistical analyses of unit-cell and lattice dynamic properties (**Supp. Fig. 6g-h**).

After ~0.04 μs, the RMSD plot showed stabilization of the central unit-cell particle (**Fig. 4g,f, Supp. Fig. 6g-j**). Analysis of pore size (the area enclosed by four-helix bundles at the vertices) after ~3 μs revealed that all pores exhibited flexibility, with the top and bottom vertices displaying the highest structural dynamics (**Fig. 4j, Supp. Fig. 7**). This observation is consistent with experimental results. The average pore sizes, ranging from 5.4 nm (horizontal pores) to 6.5 nm (top/bottom pore size), were ~27% larger than those designed or observed in conventional cryo-EM SPA maps (~ 5.1 nm). However, these pore sizes remained ~20% smaller (~1.6 nm less) than those observed in IPET 3D maps (ranging from 5.5 nm to 8.1 nm, horizontal to top/bottom pore size), suggesting a relatively stable unit-cell structure in the simulation. Further analysis showed a slight (~3%) volume decrease in the central unit-cell particle after ferritin loading, from 29,075 $nm^3$ to 28,195 $nm^3$, similar to experimental observations.

Lattice parameter and diffraction analysis: The unit-cell parameter, measured as the distance between two adjacent cages, was ~59.7 ± 2.0 nm, with a peak population distance of ~56 nm under empty and cross-linker conditions. This value was slightly larger than SAXS measurements (~57.6–58.6 nm) [2]. However, it was ~4 nm smaller than the initial lattice standard model due to lattice shrinkage caused by soft linkers. Despite this shrinkage, the simulated lattice lengths were larger than those observed experimentally, likely due to the limited simulation time or lattice size. The reduced shrinkage of the linkers also restricted the rotational and tilting freedom of the unit cells, resulting in a higher order of lattice. Simulated diffraction patterns supported this conclusion, showing relatively higher-resolution diffraction spots (**Fig. 4h**). The higher resolution indicated a more ordered lattice in the simulation compared to experiments, attributed to the reduced freedom of unit-cell rotation and tilting.

Although the simulations could not fully replicate experimental results, they exhibited similar trends, particularly in identifying flexible regions and the effects of ferritin loading. These findings demonstrate that, despite the smaller size of the simulated 5 × 5 lattice compared to experimental lattices, the consistency between simulation and experimental results validates the simulation approach as a valuable tool for evaluating unit-cell and lattice dynamics. The study highlights the utility of coarse-grained MD simulations in uncovering the structural dynamics and flexibility of lattice systems.

Strategy to stabilize the lattices: To enhance lattice order, we propose reducing structural flexibility at the top and bottom vertices of the octahedral cage. Sequence analysis and predicted secondary structures (**Supp. Fig. 1**) identified single-stranded DNA (ssDNA) segments at the distal ends of four helices near the termini of the helix bundles (HBs) at these vertices (**Extended Data Fig. 10b**). The inherent flexibility of ssDNA, coupled with its inability to maintain a stable helical structure, increases the effective loop length and introduces additional degrees of freedom between adjacent vertices, contributing to lattice disorder. To address this issue, we incorporated additional DNA staples, each comprising 42 nucleotides, to convert these ssDNA segments into double-stranded DNA (dsDNA). This modification stabilizes the helical structure at the distal ends, reducing both the length and flexibility of the loop region. As a result, this structural adjustment is expected to increase the rigidity of the cage (**Fig. 4c, Extended Data Fig. 10e**) and improve lattice order.

The second most flexible region was identified in the linkers between cages. These linkers consisted of four ssDNA strands, each 30 bases long, with only 8 base pairs hybridized to opposing linkers to connect two vertices (**Fig. 4c, Extended Data Fig. 10c**). As a result, the linkers, formed by four DNA helices, contained only ~15% dsDNA (8 base pairs out of 44 bases per strand), rendering them insufficiently strong to securely connect the two vertices, which together comprise 2 × 24 helices (**Fig. 4c, Extended Data Fig. 10f**). To address this, we redesigned the linkers to incorporate eight additional DNA staples, converting all ssDNA within the linkers into dsDNA. This increased the dsDNA content from ~15% to ~100%, significantly strengthening the inter-cage connections and potentially resulting in a higher-order lattice structure.

To validate these modifications, the redesigned cages and linkers were assembled into 5 × 5 lattices and subjected to MD simulations (**Fig. 4e,g, Supp. Fig. 6k**) as described previously. Statistical analysis of the simulations revealed reduced variability in cage volume (29,245 ± 622 $nm^3$), which aligned more closely with the designed volume (27,941 $nm^3$). The standard deviation of pore sizes across all vertices was significantly reduced (**Fig. 4j, Supp. Fig. 7**). Measurements of cage distances indicated enhanced lattice stabilization, with unit-cell parameters of 64.5 ± 1.1 nm, closely approximating the designed lattice dimensions. Simulated diffraction patterns displayed sharper diffraction spots with higher resolution and more refined details (**Fig. 4i**). Simulated SAXS data corroborated these findings, showing a notable increase in high-order peaks.

Despite these improvements in unit-cell and lattice stability, correlation analysis of lattice distortion still revealed a high correlation between lattice disorder and the combined distortions of unit-cell size and linker properties (**Supp. Fig. 8**). Nevertheless, the simulations demonstrated that introducing DNA staples to convert ssDNA to dsDNA in the linkers can substantially improve lattice order and structural stability. These results highlight the potential of strategic DNA design to enhance the structural integrity and order of DNA-based lattices.

## Discussion

In this study, we extended the application of cryo-ET to investigate unit-cell flexibility and its influence on lattice stability. Our findings highlight that strengthening the interactions at the top and bottom vertices of unit cells (cages) can mitigate dynamic behaviors and enhance the structural stability of 2D lattices. These insights have significant implications for designing more robust 2D nanomaterials and hybrid systems. Coarse-grained MD simulations validated these experimental observations, demonstrating that the presence of ssDNA staples at certain vertices contributes to increased flexibility. Introducing structural constraints in these regions could enhance the stability and functionality of DNA origami-based lattices.

Comparative analyses between IPET maps and traditional SPA methods [2] underscored the unique advantages of IPET in capturing structural diversity. SPA, constrained by symmetry assumptions and selection criteria for homogeneous particles, produces averaged structures that fail to capture the full range of 3D variability. In contrast, IPET circumvents these limitations by providing direct visualization of individual particles and their dynamic states. This capability is particularly valuable for studying mesoscopic soft materials, such as DNA origami and metal-organic frameworks (MOFs), in 2D lattice configurations.

Thermal vibrations, a universal phenomenon, play a significant role in the dynamic behavior of both hard and soft materials, with pronounced effects in 2D systems due to reduced confinement along the z-axis. While atomic-resolution tomography is achievable for hard materials [46], cryo-ET faces challenges in resolving soft biological structures at resolutions below a single nanometer due to electron dose limitations. Despite these constraints, cryo-ET/IPET delivers critical insights into the 3D structural dynamics of soft materials, enabling the exploration of design-specific variability within 2D lattices.

Unlike the well-ordered lattice structures typically revealed by conventional crystallographic approaches, cryo-ET imaging combined with IPET 3D reconstruction revealed significant 3D structural variability among unit-cell particles in low-order 2D lattices formed by octahedral DNA origami cages under various ferritin loading levels. These unit-cell particles (cages) exhibited inclinations, rotations, distortions, and asymmetric geometries that regulated lattice order. Surprisingly, ferritin loading levels did not significantly affect lattice parameters or order, as the DNA cages provided sufficient cavity space to accommodate up to two ferritin particles. These results suggest that the observed structural variability arises primarily from the inherent design of the DNA origami structure rather than protein encapsulation.

Encapsulation variability also emerged as a critical challenge. Approximately 19% of unit-cell particles encapsulated two ferritin molecules, with the positions of ferritin particles within the cage remaining unfixed. This variability underscores the need for improved precision in protein placement. Adjustments to linker positions, linker lengths, and cage dimensions (e.g., shrinking the cage to prevent dual-protein accommodation) could enhance encapsulation efficiency. Furthermore, optimizing interactions between DNA linkers and their complementary strands could improve overall stability and assembly precision.

Another challenge lies in optimizing DNA-protein conjugation for precise assembly. Some ferritin particles were found attached to the exterior of the DNA cage rather than being fully encapsulated (**Fig. 2c and 3c**). This issue likely results from nonspecific binding of residual ssDNA to proteins. Despite nine rounds of washing to remove excess and unbound DNA, some residual ssDNA may have persisted due to nonspecific interactions with the DNA origami structure. Although UV-visible analysis confirmed a reduction in free DNA, complete elimination was not achieved. These findings emphasize the challenges of optimizing DNA-protein conjugation for precision assembly.

By integrating experimental and theoretical approaches, this study provides a comprehensive analysis of the relationship between unit-cell flexibility and lattice stability in low-order arrangements. The results highlight the potential of cryo-ET/IPET to reveal 3D structural dynamics, advancing our understanding of lattice behavior. This framework offers practical strategies for improving structural order, supporting the rational design of high-order protein lattices and other nanomaterial assemblies. It also provides valuable guidelines for stabilizing and optimizing complex, low-order 2D lattice systems.

## Methods

#### *Design and synthesis of DNA-origami 2D lattice*

The octahedral DNA origami frames (referred to as Octa) were designed using the caDNAno software (http://cadnano.org/), following protocols described in previous studies [2,36,47]. The framework employed the M13mp18 single-stranded DNA (ssDNA) scaffold (Bayou Biolabs), which, with the aid of 120 ssDNA staples, formed 12 arms of the Octa structure (**Supp. Fig. 1**). Each arm comprised a six-helix bundle (HB), with two HBs consisting of 84 paired bases and four HBs remaining as ssDNA at both distal ends.

To synthesize the 2D lattice, two types of origami frames (designated as Octa I and Octa II) were designed with an additional 16 staples (72 nucleotides, nt) containing two types of lattice linkers. At one end, 42 nt hybridized with the ends of ssDNA HBs to form four equilateral vertices. At the opposite end, 8 nt containing complementary sequences (ATCCGTTA and TAACGGAT) served as lattice linkers, connecting Octa I and Octa II through vertex-to-vertex interactions, forming the Octa 2D lattice.

For ferritin encapsulation, Octa I and Octa II were modified into Octa III and Octa IV, respectively, by replacing eight staples with longer ones containing an additional 39 nt sequence (ATCCATCACTTCATACTCTACGTTGTTGTTGTTGTTGTT) to serve as protein linkers. Ferritin was also modified with an 18 nt ssDNA linker (TATGAAGTGATGGATGAT) to connect with the modified Octa frames as described [2]. These components were mixed at two molar ratios (1.35 and 0.7, protein vs. Octa) to create two variations of the Octa 2D lattice: one fully loaded with ferritin and the other partially loaded.

#### *DNA sequences of single Octa staples:*

Octa-staple-01 TCAAAGCGAACCAGACCGTTTTATATAGTC

Octa-staple-02 GCTTTGAGGACTAAAGAGCAACGGGGAGTT

Octa-staple-03 GTAAATCGTCGCTATTGAATAACTCAAGAA

Octa-staple-04 AAGCCTTAAATCAAGACTTGCGGAGCAAAT

Octa-staple-05 ATTTTAAGAACTGGCTTGAATTATCAGTGA

Octa-staple-06 GTTAAAATTCGCATTATAAACGTAAACTAG

Octa-staple-07 AGCACCATTACCATTACAGCAAATGACGGA

Octa-staple-08 ATTGCGTAGATTTTCAAAACAGATTGTTTG

Octa-staple-09 TAACCTGTTTAGCTATTTTCGCATTCATTC

Octa-staple-10 GTCAGAGGGTAATTGAGAACACCAAAATAG

Octa-staple-11 CTCCAGCCAGCTTTCCCCTCAGGACGTTGG

Octa-staple-12 GTCCACTATTAAAGAACCAGTTTTGGTTCC

Octa-staple-13 TAAAGGTGGCAACATAGTAGAAAATAATAA

Octa-staple-14 GATAAGTCCTGAACAACTGTTTAAAGAGAA

Octa-staple-15 GGTAATAGTAAAATGTAAGTTTTACACTAT

Octa-staple-16 TCAGAACCGCCACCCTCTCAGAGTATTAGC

Octa-staple-17 AAGGGAACCGAACTGAGCAGACGGTATCAT

Octa-staple-18 GTAAAGATTCAAAAGGCCTGAGTTGACCCT

Octa-staple-19 AGGCGTTAAATAAGAAGACCGTGTCGCAAG

Octa-staple-20 CAGGTCGACTCTAGAGCAAGCTTCAAGGCG

Octa-staple-21 CAGAGCCACCACCCTCTCAGAACTCGAGAG

Octa-staple-22 TTCACGTTGAAAATCTTGCGAATGGGATTT

Octa-staple-23 AAGTTTTAACGGGGTCGGAGTGTAGAATGG

Octa-staple-24 TTGCGTATTGGGCGCCCGCGGGGTGCGCTC

Octa-staple-25 GTCACCAGAGCCATGGTGAATTATCACCAATCAGAAAAGCCT

Octa-staple-26 GGACAGAGTTACTTTGTCGAAATCCGCGTGTATCACCGTACG

Octa-staple-27 CAACATGATTTACGAGCATGGAATAAGTAAGACGACAATAAA

Octa-staple-28 AACCAGACGCTACGTTAATAAAACGAACATACCACATTCAGG

Octa-staple-29 TGACCTACTAGAAAAAGCCCCAGGCAAAGCAATTTCATCTTC

Octa-staple-30 TGCCGGAAGGGGACTCGTAACCGTGCATTATATTTTAGTTCT

Octa-staple-31 AGAACCCCAAATCACCATCTGCGGAATCGAATAAAAATTTTT

Octa-staple-32 GCTCCATTGTGTACCGTAACACTGAGTTAGTTAGCGTAACCT

Octa-staple-33 AGTACCGAATAGGAACCCAAACGGTGTAACCTCAGGAGGTTT

Octa-staple-34 CAGTTTGAATGTTTAGTATCATATGCGTAGAATCGCCATAGC

Octa-staple-35 AAGATTGTTTTTAACCAAGAAACCATCGACCCAAAAACAGG

Octa-staple-36 TCAGAGCGCCACCACATAATCAAAATCAGAACGAGTAGTATG

Octa-staple-37 GATGGTTGGGAAGAAAAATCCACCAGAAATAATTGGGCTTGA

Octa-staple-38 CTCCTTAACGTAGAAACCAATCAATAATTCATCGAGAACAGA

Octa-staple-39 AGACACCTTACGCAGAACTGGCATGATTTTCTGTCCAGACAA

Octa-staple-40 GCCAGCTAGGCGATAGCTTAGATTAAGACCTTTTTAACCTGT

Octa-staple-41 CCGACTTATTAGGAACGCCATCAAAAATGAGTAACAACCCCA

Octa-staple-42 GTCCAATAGCGAGAACCAGACGACGATATTCAACGCAAGGGA

Octa-staple-43 CCAAAATACAATATGATATTCAACCGTTAGGCTATCAGGTAA

Octa-staple-44 AACAGTACTTGAAAACATATGAGACGGGTCTTTTTTAATGGA

Octa-staple-45 TTTCACCGCATTAAAGTCGGGAAACCTGATTTGAATTACCCA

Octa-staple-46 GAGAATAGAGCCTTACCGTCTATCAAATGGAGCGGAATTAGA

Octa-staple-47 ATAATTAAATTTAAAAAACTTTTTCAAACTTTTAACAACGCC

Octa-staple-48 GCACCCAGCGTTTTTTATCCGGTATTCTAGGCGAATTATTCA

Octa-staple-49 GGAAGCGCCCACAAACAGTTAATGCCCCGACTCCTCAAGATA

Octa-staple-50 GTTTGCCTATTCACAGGCAGGTCAGACGCCACCACACCACCC

Octa-staple-51 CGCGAGCTTAGTTTTTCCCAATTCTGCGCAAGTGTAAAGCCT

Octa-staple-52 AGAAGCAACCAAGCCAAAAGAATACACTAATGCCAAAACTCC

Octa-staple-53 ATTAAGTATAAAGCGGCAAGGCAAAGAAACTAATAGGGTACC

Octa-staple-54 CAGTGCCTACATGGGAATTTACCGTTCCACAAGTAAGCAGAT

Octa-staple-55 ATAAGGCGCCAAAAGTTGAGATTTAGGATAACGGACCAGTCA

Octa-staple-56 TGCTAAACAGATGAAGAAACCACCAGAATTTAAAAAAAGGCT

Octa-staple-57 CAGCCTTGGTTTTGTATTAAGAGGCTGACTGCCTATATCAGA

Octa-staple-58 CGGAATAATTCAACCCAGCGCCAAAGACTTATTTTAACGCAA

Octa-staple-59 CGCCTGAATTACCCTAATCTTGACAAGACAGACCATGAAAGA

Octa-staple-60 ACGCGAGGCTACAACAGTACCTTTTACAAATCGCGCAGAGAA

Octa-staple-61 CAGCGAACATTAAAAGAGAGTACCTTTACTGAATATAATGAA

Octa-staple-62 GGACGTTTAATTTCGACGAGAAACACCACCACTAATGCAGAT

Octa-staple-63 AAAGCGCCAAAGTTTATCTTACCGAAGCCCAATAATGAGTAA

Octa-staple-64 GAGCTCGTTGTAAACGCCAGGGTTTTCCAAAGCAATAAAGCC

Octa-staple-65 AATTATTGTTTTCATGCCTTTAGCGTCAGATAGCACGGAAAC

Octa-staple-66 AAGTTTCAGACAGCCGGGATCGTCACCCTTCTGTAGCTCAAC

Octa-staple-67 ACAAAGAAATTTAGGTAGGGCTTAATTGTATACAACGGAATC

Octa-staple-68 AACAAAAATAACTAGGTCTGAGAGACTACGCTGAGTTTCCCT

Octa-staple-69 CATAACCTAAATCAACAGTTCAGAAAACGTCATAAGGATAGC

Octa-staple-70 CACGACGAATTCGTGTGGCATCAATTCTTTAGCAAAATTACG

Octa-staple-71 CCTACCAACAGTAATTTTATCCTGAATCAAACAGCCATATGA

Octa-staple-72 GATTATAAAGAAACGCCAGTTACAAAATTTACCAACGTCAGA

Octa-staple-73 AGTAGATTGAAAAGAATCATGGTCATAGCCGGAAGCATAAGT

Octa-staple-74 TAGAATCCATAAATCATTTAACAATTTCTCCCGGCTTAGGTT

Octa-staple-75 AAAGGCCAAATATGTTAGAGCTTAATTGATTGCTCCATGAGG

Octa-staple-76 CCAAAAGGAAAGGACAACAGTTTCAGCGAATCATCATATTCC

Octa-staple-77 GAAATCGATAACCGGATACCGATAGTTGTATCAGCTCCAACG

Octa-staple-78 TGAATATTATCAAAATAATGGAAGGGTTAATATTTATCCCAA

Octa-staple-79 GAGGAAGCAGGATTCGGGTAAAATACGTAAAACACCCCCCAG

Octa-staple-80 GGTTGATTTTCCAGCAGACAGCCCTCATTCGTCACGGGATAG

Octa-staple-81 CAAGCCCCCACCCTTAGCCCGGAATAGGACGATCTAAAGTTT

Octa-staple-82 TGTAGATATTACGCGGCGATCGGTGCGGGCGCCATCTTCTGG

Octa-staple-83 CATCCTATTCAGCTAAAAGGTAAAGTAAAAAGCAAGCCGTTT

Octa-staple-84 CAGCTCATATAAGCGTACCCCGGTTGATGTGTCGGATTCTCC

Octa-staple-85 CATGTCACAAACGGCATTAAATGTGAGCAATTCGCGTTAAAT

Octa-staple-86 AGCGTCACGTATAAGAATTGAGTTAAGCCCTTTTTAAGAAAG

Octa-staple-87 TATAAAGCATCGTAACCAAGTACCGCACCGGCTGTAATATCC

Octa-staple-88 ATAGCCCGCGAAAATAATTGTATCGGTTCGCCGACAATGAGT

Octa-staple-89 AGACAGTTCATATAGGAGAAGCCTTTATAACATTGCCTGAGA

Octa-staple-90 AACAGGTCCCGAAATTGCATCAAAAAGATCTTTGATCATCAG

Octa-staple-91 ACTGCCCTTGCCCCGTTGCAGCAAGCGGCAACAGCTTTTTCT

Octa-staple-92 TCAAAGGGAGATAGCCCTTATAAATCAAGACAACAACCATCG

Octa-staple-93 GTAATACGCAAACATGAGAGATCTACAACTAGCTGAGGCCGG

Octa-staple-94 GAGATAACATTAGAAGAATAACATAAAAAGGAAGGATTAGGA

Octa-staple-95 CAGATATTACCTGAATACCAAGTTACAATCGGGAGCTATTTT

Octa-staple-96 CATATAACTAATGAACACAACATACGAGCTGTTTCTTTGGGG

Octa-staple-97 ATGTTTTGCTTTTGATCGGAACGAGGGTACTTTTTCTTTTGATAAGAGGTCATT

Octa-staple-98 GGGGTGCCAGTTGAGACCATTAGATACAATTTTCACTGTGTGAAATTGTTATCC

Octa-staple-99 CTTCGCTGGGCGCAGACGACAGTATCGGGGCACCGTCGCCATTCAGGCTGCGCA

Octa-staple-100 TCAGAGCTGGGTAAACGACGGCCAGTGCGATCCCCGTAGTAGCATTAACATCCA

Octa-staple-101 TTAGCGGTACAGAGCGGGAGAATTAACTGCGCTAATTTCGGAACCTATTATTCT

Octa-staple-102 GATATTCTAAATTGAGCCGGAACGAGGCCCAACTTGGCGCATAGGCTGGCTGAC

Octa-staple-103 TGTCGTCATAAGTACAGAACCGCCACCCATTTTCACAGTACAAACTACAACGCC

Octa-staple-104 CGATTATAAGCGGAGACTTCAAATATCGCGGAAGCCTACGAAGGCACCAACCTA

Octa-staple-105 AACATGTACGCGAGTGGTTTGAAATACCTAAACACATTCTTACCAGTATAAAGC

Octa-staple-106 GTCTGGATTTTGCGTTTTAAATGCAATGGTGAGAAATAAATTAATGCCGGAGAG

Octa-staple-107 GCCTTGAATCTTTTCCGGAACCGCCTCCCAGAGCCCAGAGCCGCCGCCAGCATT

Octa-staple-108 CGCTGGTGCTTTCCTGAATCGGCCAACGAGGGTGGTGATTGCCCTTCACCGCCT

Octa-staple-109 TGATTATCAACTTTACAACTAAAGGAATCCAAAAAGTTTGAGTAACATTATCAT

Octa-staple-110 ACATAACTTGCCCTAACTTTAATCATTGCATTATAACAACATTATTACAGGTAG

Octa-staple-111 GTAGCGCCATTAAATTGGGAATTAGAGCGCAAGGCGCACCGTAATCAGTAGCGA

Octa-staple-112 TTATTTTTACCGACAATGCAGAACGCGCGAAAAATCTTTCCTTATCATTCCAAG

Octa-staple-113 TTTCAATAGAAGGCAGCGAACCTCCCGATTAGTTGAAACAATAACGGATTCGCC

Octa-staple-114 GGGCGACCCCAAAAGTATGTTAGCAAACTAAAAGAGTCACAATCAATAGAAAAT

Octa-staple-115 AGCCGAAAGTCTCTCTTTTGATGATACAAGTGCCTTAAGAGCAAGAAACAATGA

Octa-staple-116 GTGGGAAATCATATAAATATTTAAATTGAATTTTTGTCTGGCCTTCCTGTAGCC

Octa-staple-117 CCCACGCGCAAAATGGTTGAGTGTTGTTCGTGGACTTGCTTTCGAGGTGAATTT

Octa-staple-118 ATGACCACTCGTTTGGCTTTTGCAAAAGTTAGACTATATTCATTGAATCCCCCT

Octa-staple-119 TCCAAATCTTCTGAATTATTTGCACGTAGGTTTAACGCTAACGAGCGTCTTTCC

Octa-staple-120 GGGTTATTTAATTACAATATATGTGAGTAATTAATAAGAGTCAATAGTGAATTT

***DNA sequences of lattice linker:***

To form 2D lattice, the following DNA sequences were added.

**Octa I:**

CTTCATCAAGAGAAATCAACGTAACAGAGATTTGTCAATCATTTTTTTTTTTTTTTTTTTTTTTATCCGTTA

AAAGATTCATCAGGAATTACGAGGCATGCTCATCCTTATGCGTTTTTTTTTTTTTTTTTTTTTTATCCGTTA

ATAAATCATACATAAATCGGTTGTACTGTGCTGGCATGCCTGTTTTTTTTTTTTTTTTTTTTTTATCCGTTA

GGTAGCTATTTTAGAGAATCGATGAAAACATTAAATGTGTAGTTTTTTTTTTTTTTTTTTTTTTATCCGTTA

CAAATGCTTTAAAAAATCAGGTCTTTAAGAGCAGCCAGAGGGTTTTTTTTTTTTTTTTTTTTTTATCCGTTA

CAACGCTCAACAGCAGAGGCATTTTCAATCCAATGATAAATATTTTTTTTTTTTTTTTTTTTTTATCCGTTA

AGCTTTCATCAACGGATTGACCGTAAAATCGTATAATATTTTTTTTTTTTTTTTTTTTTTTTTTATCCGTTA

AGAGCCTAATTTGATTTTTTGTTTAAATCCTGAAATAAAGAATTTTTTTTTTTTTTTTTTTTTATCCGTTA

AAACGAAAGAGGGCGAAACAAAGTACTGACTATATTCGAGCTTTTTTTTTTTTTTTTTTTTTTTATCCGTTA

ACTGTTGGGAAGCAGCTGGCGAAAGGATAGGTCAAGATCGCATTTTTTTTTTTTTTTTTTTTTTATCCGTTA

AACGGGTATTAAGGAATCATTACCGCCAGTAATTCAACAATATTTTTTTTTTTTTTTTTTTTTTATCCGTTA

GAAACATGAAAGCTCAGTACCAGGCGAAAAATGCTGAACAAATTTTTTTTTTTTTTTTTTTTTTATCCGTTA

ATCAAAATCATATATGTAAATGCTGAACAAACACTTGCTTCTTTTTTTTTTTTTTTTTTTTTTTATCCGTTA

TGATTGCTTTGAGCAAAAGAAGATGAAATAGCAGAGGTTTTGTTTTTTTTTTTTTTTTTTTTTTATCCGTTA

TTTGCGGAACAATGGCAATTCATCAATCTGTATAATAATTTTTTTTTTTTTTTTTTTTTTTTTATCCGTTA

TGTAGCATTCCAACGTTAGTAAATGAAGTGCCGCGCCACCCTTTTTTTTTTTTTTTTTTTTTTTATCCGTTA

Octa II:

CTTCATCAAGAGAAATCAACGTAACAGAGATTTGTCAATCATTTTTTTTTTTTTTTTTTTTTTTAACGGAT

AAAGATTCATCAGGAATTACGAGGCATGCTCATCCTTATGCGTTTTTTTTTTTTTTTTTTTTTTAACGGAT

ATAAATCATACATAAATCGGTTGTACTGTGCTGGCATGCCTGTTTTTTTTTTTTTTTTTTTTTTTAACGGAT

GGTAGCTATTTTAGAGAATCGATGAAAACATTAAATGTGTAGTTTTTTTTTTTTTTTTTTTTTTTAACGGAT

CAAATGCTTTAAAAAATCAGGTCTTTAAGAGCAGCCAGAGGGTTTTTTTTTTTTTTTTTTTTTTTAACGGAT

CAACGCTCAACAGCAGAGGCATTTTCAATCCAATGATAAATATTTTTTTTTTTTTTTTTTTTTTTAACGGAT

AGCTTTCATCAACGGATTGACCGTAAAATCGTATAATATTTTTTTTTTTTTTTTTTTTTTTTTTTAACGGAT

AGAGCCTAATTTGATTTTTTGTTTAAATCCTGAAATAAAGAATTTTTTTTTTTTTTTTTTTTTTAACGGAT

AAACGAAAGAGGGCGAAACAAAGTACTGACTATATTCGAGCTTTTTTTTTTTTTTTTTTTTTTTTAACGGAT

ACTGTTGGGAAGCAGCTGGCGAAAGGATAGGTCAAGATCGCATTTTTTTTTTTTTTTTTTTTTTTAACGGAT

AACGGGTATTAAGGAATCATTACCGCCAGTAATTCAACAATATTTTTTTTTTTTTTTTTTTTTTTAACGGAT

GAAACATGAAAGCTCAGTACCAGGCGAAAAATGCTGAACAAATTTTTTTTTTTTTTTTTTTTTTTAACGGAT

ATCAAAATCATATATGTAAATGCTGAACAAACACTTGCTTCTTTTTTTTTTTTTTTTTTTTTTTTAACGGAT

TGATTGCTTTGAGCAAAAGAAGATGAAATAGCAGAGGTTTTGTTTTTTTTTTTTTTTTTTTTTTTAACGGAT

TTTGCGGAACAATGGCAATTCATCAATCTGTATAATAATTTTTTTTTTTTTTTTTTTTTTTTTTTAACGGAT

TGTAGCATTCCAACGTTAGTAAATGAAGTGCCGCGCCACCCTTTTTTTTTTTTTTTTTTTTTTTTAACGGAT

#### *Cryo-EM specimen preparation*

Three types of DNA origami 2D lattice samples were prepared for observation: (1) a 100% loading ferritin-encapsulated origami lattice (1.35× molar ratio of origami to protein), (2) a 70% loading ferritin-encapsulated origami lattice (0.7× molar ratio of origami to protein), and (3) an Octa lattice without ferritin loading. Cryo-EM technique was employed for specimen preparation. In brief, a 4 μL aliquot of the sample was placed on a lacey carbon film-coated EM grid that was also glow-discharged for 15 seconds. After blotting for 3.5 seconds, the grid was immediately plunged into liquid ethane using a Leica EM GP plunge freezer under controlled conditions (~90% humidity and 4°C).

#### *Cryo-EM and cryo-ET data acquisition*

For 100% and 0% loading ferritin-encapsulated samples, imaging was performed using a Titan Krios G2 TEM equipped with a Gatan energy filter, operated at 300 keV. Micrographs were recorded on a Gatan K3 direct electron detector in super-resolution mode at a magnification of 53,000×, corresponding to 1.46 Å/pixel, with a defocus of ~3 μm. Using SerialEM [48], the tomography sets were collected over a range of -51° to +51° in 3° increments with two exposure conditions as below: 1) tomo 1 for 100% loading lattice: 2.2-second exposure time at a dose rate of ~3 $e^{-}Å^{-2}s^{-1}$, and the total dose is ~231 $e^{-}Å^{-2}$; 2) tomo 2 for 100% loading lattice and tomo 4 for 0% loading lattice: 1.0-second exposure time at a dose rate of ~6 $e^{-}Å^{-2}s^{-1}$, and the total dose is ~210 $e^{-}Å^{-2}$. The un-tilted micrographs were acquired with a total dose of ~50 $e^{-}Å^{-2}$.For 70% loading ferritin-encapsulated origami 2D lattice specimens, imaging was conducted on a Zeiss Libra 120 Plus TEM with a 4k × 4k Gatan UltraScan CCD, using a Gatan 626 cryo-holder. The TEM was operated at 120 kV in low-dose mode. Tilt series of tomo 3 were acquired at a magnification of 80,000×, corresponding to 1.48 Å/pixel, with an angle range from -48° to +48° in 3° increments, at a defocus of ~7 μm, using the Gatan tomography software package and a custom-developed fully automated ET software [27]. The micrographs were taken with a 1.0-second exposure, and the total dose per tilt series is ~66 $e^{-}Å^{-2}$.

#### *Image preprocessing*

Motion correction for multi-frame images captured by the K3 camera was performed using MotionCor2 [49], a critical step to enhance image quality prior to further processing. The tilt series of entire micrographs were subsequently aligned using IMOD [41], ensuring consistency across the series for accurate 3D reconstruction. The Contrast Transfer Function (CTF) was determined using the GCTF software package [50] and corrected with TOMOCTF [51].

To further reduce image noise, selected micrographs were processed using a custom-developed machine learning software (manuscript in preparation), as well as median-filter software and a contrast enhancement method previously developed [26]. These steps collectively improved the quality of data for downstream analysis.

#### *3D reconstruction process*

The 3D reconstruction of the entire lattice was performed using IMOD software[41] after binning the tilt series eight times, resulting in pixel sizes of 11.68 Å for K3 images and 11.84 Å for CCD images. High-resolution 3D structures of individual DNA origami particles within the 2D lattice were reconstructed independently using the IPET method with focused 3D reconstruction algorithms [25]. This process began by isolating each targeted origami particle from the tilt series of approximately 256 × 256 pixels, corresponding to a pixel size of 2.92 Å for K3 images and 2.96 Å for CCD images.

An initial 3D model for each smaller series was generated via back-projection, which initiated the 3D reconstruction process. During the iterative refinement stages, Gaussian low-pass filters, soft-boundary circular masks, and particle-shaped masks were applied to enhance the signal-to-noise ratio (SNR). To address artifacts from the limited tilt angle range, the final 3D maps were corrected for the missing wedge effect using the LoTToR method [27].

#### *Resolution estimation*

The resolution of the final 3D reconstructions was determined using Fourier Shell Correlation (FSC), which compared two 3D maps generated from odd and even tilt-series indices[25]. The frequencies at which the FSC curve dropped to 0.143 or 0.5 were used to define the resolution of the 3D density maps [52]. Additionally, map-model-based FSC analysis was performed, where the FSC curve was calculated between the final IPET reconstruction and the map generated from its fitting model, with the frequency at FSC=0.5 serving as the estimated resolution.

To validate the FSC-estimated resolution, observable structural features were examined. These features included individual helices (~2 nm in diameter), the 6-HBs (~6–8 nm in diameter), ferritin (~12 nm in diameter), and the octahedral cage (~60 nm in diameter). The clear visualization of the cage, ferritin, 6-HBs, and even individual rod-like helices suggested that the resolution reached approximately 2–3 nm. To analyze the flexibility of the cage, all final IPET reconstructions were low-pass filtered to 80 Å using EMAN software [53].

#### *Modelling the 3D structure of individual unit-cell particle*

To create a standardized 3D structure template for refining the structure of each unit-cell particle, the model was generated using the oxDNA viewer [44], a browser-based tool designed for visualizing DNA structures through rigid cluster dynamics. The process began with converting a flat CaDNAno design file [54] into topology and configuration files using TacoxDNA software [55]. The resulting standard model from oxDNA exhibited octahedral symmetry, featuring 12 arms (or helices) each measuring 28.5 nm. This model was subsequently converted into a Protein Data Bank (PDB) file via TacoxDNA, facilitating the generation of a density map using the pdb2mrc command in the EMAN software suite [53].

From this density map, a single arm was extracted as a standard arm map. To integrate these models into the IPET 3D density map, 12 copies of the standard arm map were aligned and superimposed using the fitmap operation in UCSF Chimera [42], optimizing overlap between the reconstructed models and their corresponding arm positions. The spatial coordinates of the two distal ends of each arm in the best-fitted map were used as reference points for further refinement and repositioning of the arms in the standard model via a custom Python script.

To further refine the docking model, the structure was subjected to a relaxation simulation in oxDNA for 5,000 steps (~15.15 ns) using an integration time step of 0.005 (~3.03 ps per step). The resulting topology file from the simulation was converted back into a PDB file using TacoxDNA, yielding a flexibly fitted structure tailored to the targeted IPET map.

For docking the ferritin model, the central location of ferritin within the IPET 3D maps was identified by pinpointing the highest-density region in the ferritin portion of the map relative to the surrounding particle density. While this method enabled precise placement and structural characterization of ferritin within the particle model, it was not reliable for determining the orientation of ferritin.

#### *Molecular dynamic simulations*

For molecular dynamics (MD) simulations, a 5 × 5 lattice consisting of 25 octahedral models was constructed using oxDNA and oxView [45,56]. This lattice size was selected to facilitate a comparative analysis of structural differences between octahedral models near the boundary and those at the center. Two model variations, octahedral model I and model II, were used as described earlier [2]. Due to undefined connections between model I and II, two types of 5 × 5 lattices were constructed for this study, with linkers arranged in either parallel or crossed configurations. Prior to MD simulation, these lattices underwent structural relaxation, including energy minimization over 300,000 steps in oxDNA.

The MD simulations were conducted with each lattice type placed into a cubic box measuring 426 nm across, filled with a salt concentration of $[Na^+]$ = 0.5 M to approximate the experimental salt concentration of 12.5 mM. A Langevin-like dynamics algorithm was employed to simulate solvent effects, inducing Brownian motion in the nucleotides [56]. The 5 × 5 lattice model was simulated for over 2 × $10^8$ steps (equivalent to 0.606 ms duration) at a temperature of 277 K. Lattice stability over time was assessed through root mean square deviation (RMSD) analysis, providing insights into the dynamic behavior and structural integrity of the lattice.

To model the 2D lattice with ferritin, octahedral model III and model IV units were modified by incorporating 8 nt ssDNA linkers within each model cage. These modified units were assembled into two types of 5 × 5 lattices with either parallel or crossing vertex-to-vertex connections. For MD simulations investigating the impact of ferritin on the lattice, the ferritin molecules were modeled as rigid-body spherical objects. During the simulations, the distances between the protein linkers ("T" nts on the 8 ssDNA linkers) were maintained constant using a mutual trap method [56].

Lattice flexibility was analyzed by selecting MD snapshots at intervals of 50,000 steps following system equilibration. For each snapshot, 24 coordinates representing the vertices (i.e., the distal ends of the 12 arms within each octahedral model) were extracted to characterize the structure and conformation of the octahedral model, in a manner analogous to the experimental results. Using these coordinates, the geometric center and volume of each octahedral model were calculated.

The lattice unit parameters were then determined for both the entire 5 × 5 lattice and the central 3 × 3 section, providing insights into the dynamic behavior of the lattice and the effects of ferritin encapsulation.

#### RMSD Analysis

Root Mean Square Deviation (RMSD) analysis between two models was conducted by aligning them using least-squares fitting, executed via the *match* command in UCSF Chimera. For hierarchical clustering of the RMSD values, the *scipy.cluster.hierarchy.linkage* function in Python was utilized. This analysis involved calculating RMSD values for each pair of models within a set of IPET models. The distance between a newly formed cluster, , and each existing cluster, , was computed using the Ward variance minimization method. The new entry , was calculated using the following equation:

$$d(u,v) = \sqrt{\frac{|v|+|s|}{T} d(v,s)^2 + \frac{|v|+|t|}{T} d(v,t)^2 - \frac{|v|}{T} d(s,t)^2} \quad (1)$$

where $u$ is the newly joined cluster consisting of clusters $s$ and $t$, $v$ is an unused cluster in the forest, and $T = |v| + |s| + |t|$. To visualize the results of the hierarchical clustering, a dendrogram was generated, illustrating the clustering relationships based on the RMSD values.

#### Strategy for repair the low-ordered 2D lattice

Given that the highest diffraction spots observed correspond to a resolution of ~18 nm, traditional crystallographic methods are unable to achieve resolutions beyond this limit. This is insufficient to resolve the detailed structure of the unit-cell particle, which consists of 12 arms, each with a diameter of

~5.6 nm (comprising six-helix bundles, 6HBs). More critically, an averaged map of unit-cell particles fails to capture the structural variability that contributes to lattice flexibility and low ordering.

To address this challenge, our strategy involves two key steps: i) Low-resolution reconstruction of the entire lattice: A low-resolution 3D density map of the entire lattice was reconstructed using IMOD software [41] by aligning cryo-ET micrographs after CTF correction[25]. ii) High-resolution reconstruction of individual unit-cell particles: High-resolution 3D density maps of each unit-cell particle were reconstructed using IPET[51] through iterative alignment of cropped images of individual unit-cell particles extracted from the initially aligned large micrographs.

To integrate these reconstructions, the low-resolution IMOD map was used as a global constraint to define the locations and orientations of the unit-cell particles. High-resolution IPET maps were then aligned to their respective unit-cell particles through local cross-correlation calculations and integrated to produce a high-resolution map of the entire lattice.

The final resolution was assessed using three independent methods [52]: i) Map-to-map method: The frequency of the FSC curve between two half-reconstructions (derived from even and odd tilt image indices) [57] was used to estimate resolution. ii) Map-to-model method [57]: The FSC frequency between the IPET map and its fitted structural model provided an additional resolution estimate. iii) Feature-based evaluation: The resolution was evaluated by assessing observable structural features in the 3D map, including the ~12 nm ferritin, the 5.6 nm diameter 6HB arms, the ~2 nm diameter dsDNA helix, and the DNA helix ~1.2 nm major and ~0.5 nm minor grooves. This combined approach effectively reconstructs the 3D structure of low-ordered 2D lattices while capturing the structural variability of unit-cell particles, providing insights into lattice flexibility and order.

#### *Statistical analyses of lattice and unit-cell particle structures*

The structural analysis of the 2D lattice was conducted at three levels: (i) the lattice, (ii) interactions between unit-cell particles, and (iii) the internal structure of an individual unit-cell particle.

Lattice-Level Analysis: Each unit-cell particle was represented by its central point, defined as the average of the coordinates of the 24 distal ends of the 12 fitting arms within the unit-cell particle. The optimal model for each arm was determined by rigid-body alignment of the arm structure to the experimental density map of the corresponding arm, followed by flexible fitting using MD simulations. Measurements included: The distances between every two adjacent unit-cell particle central points, and the angles formed by every three-consecutive unit-cell particle centers.

To evaluate the displacement of unit-cell particle centers relative to the lattice, a best-fit plane was generated based on the measured centers. Standard unit-cell particle centers were derived using the average distances between adjacent centers and the mean interaxial angle. Lattice distortions, such as ripples or crumples, were visualized by aligning the lattice with the X and Y axes and measuring the displacement between observed and standard unit-cell particle centers.

Inter-unit-cell analysis: a) In-plane rotation angles: Determined by analyzing the centers of the four in-plane vertices (calculated as the average coordinates of the four distal ends of the arms) relative to the fitted lattice plane; b) Tilting angles: Measured by assessing the orientation of the axis connecting two off-plane vertices relative to the fitted lattice plane.

Intra-unit-cell particle analysis: a) A total of 15 vertex-to-vertex distances were measured, including 12 distances along the arms and 3 along the diagonals; b) The volume of each unit-cell particle was calculated as the volume of a polyhedron formed by its six vertices; c) Two diagonal angles and two dihedral angles within the quadrilateral formed by the four in-plane vertices were measured, and correlations between angle pairs were analyzed; c) To assess unit-cell particle flexibility, all fitted models were aligned using RMSD and analyzed through hierarchical clustering to identify conformational variations.

#### Correlation analyses between lattice distortion and unit distortion

To investigate the relationship between unit-to-unit distances and changes in the distances between diagonal vertices within units, scatter plots were generated comparing the lattice unit lengths along the X- and Y-axes with the distances between two diagonal vertices along the corresponding axis in the lattice plane. The degree of variation between these two lengths was quantified by calculating the correlation coefficient (R value).

Additionally, the relationship between bending angles at lattice points and dihedral angles within units was analyzed. The bending angles at lattice points were determined by measuring the angle formed by three neighboring lattice points along one direction of the lattice. The dihedral angles within a unit were calculated by measuring the angle between two planes within an octahedron. These planes were defined by the axes connecting two off-plane vertices to each of two in-plane vertices along the same lattice direction.

The correlation between bending angles and dihedral angles was quantified using the R value, providing a measure of how changes in lattice geometry are linked to internal unit distortions. This analysis elucidates the interplay between unit flexibility and overall lattice stability.

## Conclusion

This study demonstrates the power of cryo-ET/IPET in resolving the 3D structural dynamics of low-order 2D lattices, providing unprecedented insights into unit-cell flexibility, lattice disorder, and biomolecular assembly precision. By correlating unit-cell flexibilities with lattice stability, we identified key design parameters that influence long-range order. These findings pave the way for rational engineering of high-order protein lattices, self-assembled DNA nanomaterials, and hybrid biomolecular architectures. Moving forward, incorporating molecular-level design optimizations, such as sequence modifications for enhanced rigidity and linker engineering for controlled assembly, will be critical for translating these principles into scalable, functional materials. This framework offers a blueprint for future advances in bio-nanotechnology, guiding the precise control of self-assembling molecular systems for diverse applications in synthetic biology, drug delivery, and nanoengineering.

## Declarations

#### Data availability

A total of 337 IPET 3D density maps reconstructed from each individual DNA-origami unit-cell particle in this study have been deposited in the EMDB under the following accession codes: **EMD-46070** to **EMD-46214** (for 100% ferritin loading), **EMD-46215** to **EMD-46284** (for 70% ferritin loading), and **EMD-46285** to **EMD-46406** (for 0% loading), **EMD-49283** and **EMD-49285** (for the two arms of particle #001 with 100% ferritin loading). The IMOD 3D reconstructions of the entire lattices loaded with 100%, 70% and 0% ferritin were deposited under the accession codes **EMD-49286**, **EMD-49287**, **EMD-49288** and **EMD-49289**. The associated cryo-ET raw tilt series of the lattices and their containing unit-cell particles, final maps, fitting models, and EMDB reports have been uploaded to a Figshare repository at: **https://doi.org/10.6084/m9.figshare.xxxxxx**. The process of IPET 3D reconstructions from each individual unit-cell particle, including the raw tilted images, the projections of the intermediates of 3D reconstruction, the final reconstruction and its fitting model at seven representative tilt angles are showed in **Supp. Figs. 9-345**. The measured angles on each unit-cell particle within these particles are detailed in **Supp. Table 1** and all measured data used for producing the curves in figures are available in the Source Data file.

#### Acknowledgement

We extend our gratitude Drs. Dongsheng Lei, Tom Goddard, Petr Sulc and Jonathan Doye for their invaluable discussion; We also thank Dr. Dan Toso at Cal-Cryo-EM center of QB3-Berkeley for his support with the cryo-EM/cryo-ET imaging. The work at the molecular foundry, LBNL, was supported by the Office of Science, Office of Basic Energy Sciences of the United States Department of Energy (contract no. DE-AC02-05CH11231), and US National Institutes of Health grants R01HL115153, R01GM104427, R01MH077303, R01DK042667 (GR, JL, MZ).

#### Author contributions

This project was initiated and designed by OG and GR. STW and OG prepared all DNA origami samples. JL handled the preparation of the TEM samples, data acquisition and processing, 3D density map reconstruction, and model docking and analysis, with MZ, ZH and HW contributing to the contrast enhancement by developing the machine learning program. JF carried out the MD simulations. JL and GR interpreted and refined the structural data, and drafted the initial manuscript, which was revised by GR, STW, MZ, ZH, HW and OG, ensuring a collaborative effort in the final publication.

#### Competing financial interests

The authors declare no competing financial interests.

## References

1. Yeates TO, Liu Y, Laniado J (2016) The design of symmetric protein nanomaterials comes of age in theory and practice. Curr Opin Struct Biol 39:134–143. 10.1016/j.sbi.2016.07.003
2. Wang ST et al (2021) Designed and biologically active protein lattices. Nat Commun 12:3702. 10.1038/s41467-021-23966-4
3. Ben-Sasson AJ et al (2021) Design of biologically active binary protein 2D materials. Nature 589:468–473. 10.1038/s41586-020-03120-8
4. Gonen S, DiMaio F, Gonen T, Baker D (2015) Design of ordered two-dimensional arrays mediated by noncovalent protein-protein interfaces. Science 348:1365–1368. 10.1126/science.aaa9897
5. Chen Z et al (2019) Self-Assembling 2D Arrays with de Novo Protein Building Blocks. J Am Chem Soc 141:8891–8895. 10.1021/jacs.9b01978
6. Bujold KE, Lacroix A, Sleiman HF (2018) DNA Nanostructures at the Interface with Biology. Chem 4:495–521. 10.1016/j.chempr.2018.02.005
7. Altug H, Oh SH, Maier SA, Homola J (2022) Advances and applications of nanophotonic biosensors. Nat Nanotechnol 17:5–16. 10.1038/s41565-021-01045-5
8. Kershner RJ et al (2009) Placement and orientation of individual DNA shapes on lithographically patterned surfaces. Nat Nanotechnol 4:557–561. 10.1038/nnano.2009.220
9. Maune HT et al (2010) Self-assembly of carbon nanotubes into two-dimensional geometries using DNA origami templates. Nat Nanotechnol 5:61–66. 10.1038/nnano.2009.311
10. Kuzyk A, Jungmann R, Acuna GP, Liu N (2018) DNA Origami Route for Nanophotonics. ACS Photonics 5:1151–1163. 10.1021/acsphotonics.7b01580
11. Laughlin TG et al (2022) Architecture and self-assembly of the jumbo bacteriophage nuclear shell. Nature 608:429–435. 10.1038/s41586-022-05013-4
12. Martynenko IV et al (2023) Site-directed placement of three-dimensional DNA origami. Nat Nanotechnol 18:1456–1462. 10.1038/s41565-023-01487-z
13. Nannenga BL, Gonen T (2019) The cryo-EM method microcrystal electron diffraction (MicroED). Nat Methods 16:369–379. 10.1038/s41592-019-0395-x
14. Henderson R et al (1990) Model for the structure of bacteriorhodopsin based on high-resolution electron cryo-microscopy. J Mol Biol 213:899–929. 10.1016/S0022-2836(05)80271-2
15. Kuhlbrandt W, Wang DN, Fujiyoshi Y (1994) Atomic model of plant light-harvesting complex by electron crystallography. Nature 367:614–621. 10.1038/367614a0
16. Ren G, Reddy VS, Cheng A, Melnyk P, Mitra AK (2001) Visualization of a water-selective pore by electron crystallography in vitreous ice. Proc Natl Acad Sci USA 98:1398–1403. 10.1073/pnas.98.4.1398
17. Gonen T et al (2005) Lipid-protein interactions in double-layered two-dimensional AQP0 crystals. Nature 438:633–638. 10.1038/nature04321

18. Righetto RD, Biyani N, Kowal J, Chami M, Stahlberg H (2019) Retrieving high-resolution information from disordered 2D crystals by single-particle cryo-EM. Nat Commun 10:1722. 10.1038/s41467-019-09661-5
19. Keen DA, Goodwin AL (2015) The crystallography of correlated disorder. Nature 521:303–309. 10.1038/nature14453
20. Nogales E, Mahamid J (2024) Bridging structural and cell biology with cryo-electron microscopy. Nature 628:47–56. 10.1038/s41586-024-07198-2
21. Liu J et al (2024) Non-averaged single-molecule tertiary structures reveal RNA self-folding through individual-particle cryo-electron tomography. Nat Commun 15:9084. 10.1038/s41467-024-52914-1
22. Xue L et al (2022) Visualizing translation dynamics at atomic detail inside a bacterial cell. Nature 610:205–211. 10.1038/s41586-022-05255-2
23. Bharat TA, Scheres SH (2016) Resolving macromolecular structures from electron cryo-tomography data using subtomogram averaging in RELION. Nat Protoc 11:2054–2065. 10.1038/nprot.2016.124
24. Rangan R et al (2024) CryoDRGN-ET: deep reconstructing generative networks for visualizing dynamic biomolecules inside cells. Nat Methods 21:1537–1545. 10.1038/s41592-024-02340-4
25. Zhang L, Ren G (2012) IPET and FETR: experimental approach for studying molecular structure dynamics by cryo-electron tomography of a single-molecule structure. PLoS ONE 7:e30249. 10.1371/journal.pone.0030249
26. Wu H et al (2018) An Algorithm for Enhancing the Image Contrast of Electron Tomography. Sci Rep 8:16711. 10.1038/s41598-018-34652-9
27. Zhai X et al (2020) LoTToR: An Algorithm for Missing-Wedge Correction of the Low-Tilt Tomographic 3D Reconstruction of a Single-Molecule Structure. *Scientific reports* 10, 10489. 10.1038/s41598-020-66793-1
28. Liu J et al (2016) Fully Mechanically Controlled Automated Electron Microscopic Tomography. Sci Rep 6:29231. 10.1038/srep29231
29. Lei D et al (2019) Single-molecule 3D imaging of human plasma intermediate-density lipoproteins reveals a polyhedral structure. Biochim et Biophys acta Mol cell biology lipids 1864:260–270. 10.1016/j.bbalip.2018.12.004
30. Lei D et al (2018) Three-dimensional structural dynamics of DNA origami Bennett linkages using individual-particle electron tomography. Nat Commun 9:592. 10.1038/s41467-018-03018-0
31. Zhang L et al (2016) Three-dimensional structural dynamics and fluctuations of DNA-nanogold conjugates by individual-particle electron tomography. Nat Commun 7:11083. 10.1038/ncomms11083
32. Yu Y et al (2016) Polyhedral 3D structure of human plasma very low density lipoproteins by individual particle cryo-electron tomography1. J Lipid Res 57:1879–1888. 10.1194/jlr.M070375
33. Henderson R (2004) Realizing the potential of electron cryo-microscopy. Q Rev Biophys 37:3–13. 10.1017/s0033583504003920

34. Tian Y et al (2016) Lattice engineering through nanoparticle-DNA frameworks. Nat Mater 15:654–661. 10.1038/nmat4571
35. Tian Y et al (2015) Prescribed nanoparticle cluster architectures and low-dimensional arrays built using octahedral DNA origami frames. Nat Nanotechnol 10:637–644. 10.1038/nnano.2015.105
36. Tian Y et al (2020) Ordered three-dimensional nanomaterials using DNA-prescribed and valence-controlled material voxels. Nat Mater 19:789–796. 10.1038/s41563-019-0550-x
37. Michelson A et al (2022) Three-dimensional visualization of nanoparticle lattices and multimaterial frameworks. Science 376:203–207. 10.1126/science.abk0463
38. Granier T, Gallois B, Dautant A, Langlois d'Estaintot B, Precigoux G (1997) Comparison of the structures of the cubic and tetragonal forms of horse-spleen apoferritin. Acta Crystallogr D Biol Crystallogr 53:580–587. 10.1107/S0907444997003314
39. Veneziano R et al (2016) Designer nanoscale DNA assemblies programmed from the top down. Science 352:1534. 10.1126/science.aaf4388
40. Jun H et al (2019) Automated Sequence Design of 3D Polyhedral Wireframe DNA Origami with Honeycomb Edges. ACS Nano 13:2083–2093. 10.1021/acsnano.8b08671
41. Kremer JR, Mastronarde DN, McIntosh JR (1996) Computer visualization of three-dimensional image data using IMOD. J Struct Biol 116:71–76. 10.1006/jsbi.1996.0013
42. Pettersen EF et al (2004) UCSF Chimera–a visualization system for exploratory research and analysis. J Comput Chem 25:1605–1612. 10.1002/jcc.20084
43. Fasolino A, Los JH, Katsnelson MI (2007) Intrinsic ripples in graphene. Nat Mater 6:858–861. 10.1038/nmat2011
44. Poppleton E et al (2020) Design, optimization and analysis of large DNA and RNA nanostructures through interactive visualization, editing and molecular simulation. Nucleic Acids Res 48:e72. 10.1093/nar/gkaa417
45. Bohlin J et al (2022) Design and simulation of DNA, RNA and hybrid protein-nucleic acid nanostructures with oxView. Nat Protoc 17:1762–1788. 10.1038/s41596-022-00688-5
46. Miao J, Ercius P, Billinge SJ (2016) Atomic electron tomography: 3D structures without crystals. Science 353. 10.1126/science.aaf2157
47. Liu W, Halverson J, Tian Y, Tkachenko AV, Gang O (2016) Self-organized architectures from assorted DNA-framed nanoparticles. Nat Chem 8:867–873. 10.1038/nchem.2540
48. Mastronarde DN (2005) Automated electron microscope tomography using robust prediction of specimen movements. J Struct Biol 152:36–51. 10.1016/j.jsb.2005.07.007
49. Zheng SQ et al (2017) MotionCor2: anisotropic correction of beam-induced motion for improved cryo-electron microscopy. Nat Methods 14:331–332. 10.1038/nmeth.4193
50. Zhang K, Gctf (2016) Real-time CTF determination and correction. J Struct Biol 193:1–12. 10.1016/j.jsb.2015.11.003

51. Fernandez JJ, Li S, Crowther RA (2006) CTF determination and correction in electron cryotomography. Ultramicroscopy 106:587–596. 10.1016/j.ultramic.2006.02.004
52. Lawson CL et al (2021) Cryo-EM model validation recommendations based on outcomes of the 2019 EMDataResource challenge. Nat Methods 18:156–164. 10.1038/s41592-020-01051-w
53. Ludtke SJ, Baldwin PR, Chiu W (1999) EMAN: semiautomated software for high-resolution single-particle reconstructions. J Struct Biol 128:82–97. 10.1006/jsbi.1999.4174
54. Douglas SM et al (2009) Rapid prototyping of 3D DNA-origami shapes with caDNAno. Nucleic Acids Res 37:5001–5006. 10.1093/nar/gkp436
55. Suma A et al (2019) TacoxDNA: A user-friendly web server for simulations of complex DNA structures, from single strands to origami. J Comput Chem 40:2586–2595. 10.1002/jcc.26029
56. Snodin BE et al (2015) Introducing improved structural properties and salt dependence into a coarse-grained model of DNA. J Chem Phys 142:234901. 10.1063/1.4921957
57. Ward JH (1963) Hierarchical Grouping to Optimize an Objective Function. J Am Stat Assoc 58:236–244. 10.1080/01621459.1963.10500845
58. Scheres SH, Chen S (2012) Prevention of overfitting in cryo-EM structure determination. Nat Methods 9:853–854. 10.1038/nmeth.2115

## Figures

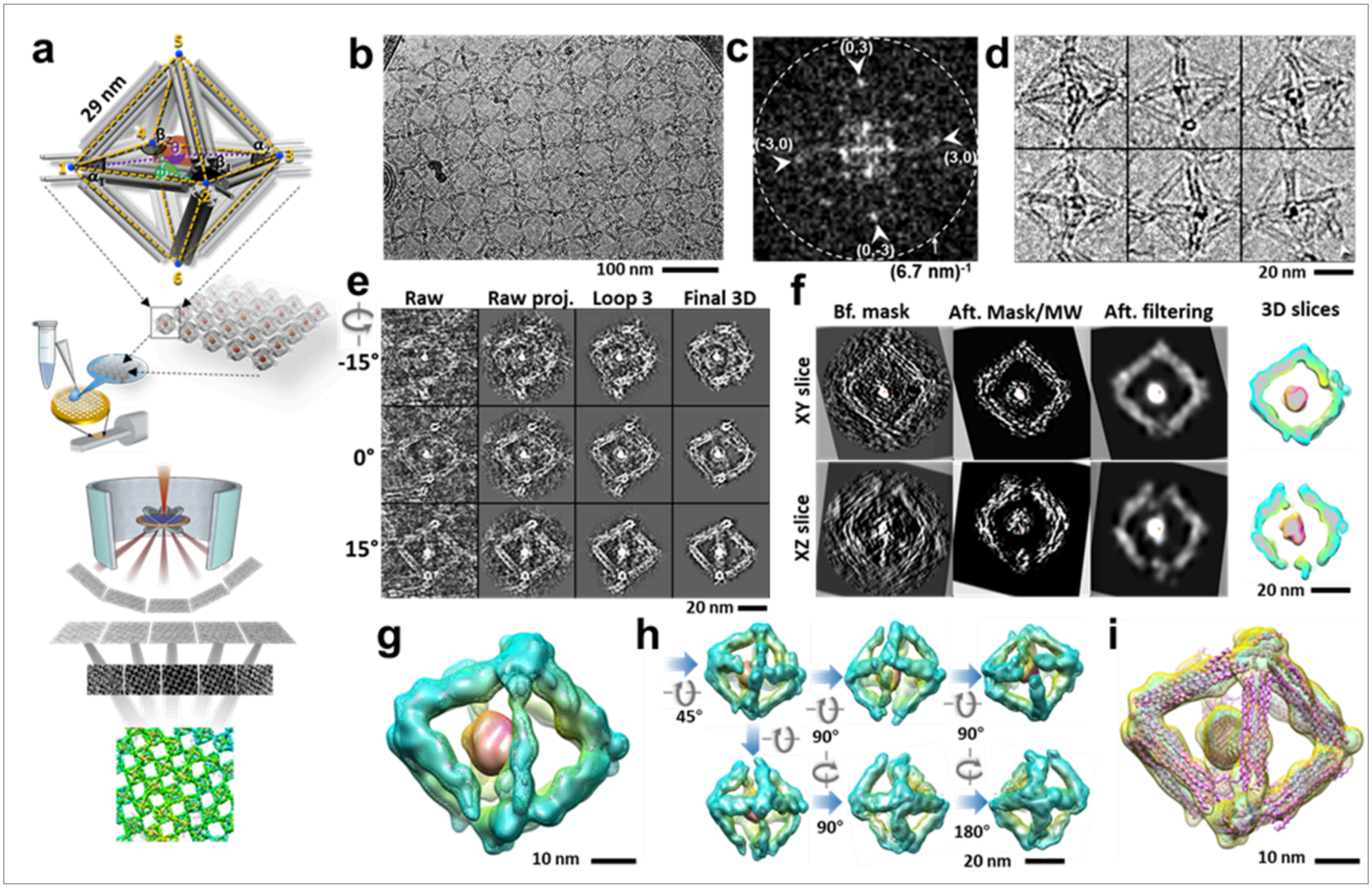

### Figure 1

**Non-averaged single unit-cell 3D structure within the 2D lattice of ferritin-DNA-origami revealed by cryo-ET and IPET. a,** Schematic representation of cryo-ET imaging and reconstruction of DNA origami 2D lattice. Each unit-cell particle incorporates a ferritin protein at its core and is surrounded by a 12-helix bundleconsisting 6 DNA helices. **b**, Cryo-EM micrograph of a 2D lattice embedded in vitrified ice, **c**, Fourier transform pattern of the lattice micrograph. **d**, Six representative unit-cell particles within the lattice. **e**, The process of IPET 3D reconstruction of a single unit-cell particles, including its intermediates and final map, presented from three representative tilt angles. **f**, Two perpendicular cross-section views of the IPET 3D reconstruction intermediates and final map, displayed by before and after the application of soft-masks and missing-wedge correction, and low-pass filtering to 60 Å resolution. The center slices of the final map are also showed from two corresponding viewing angles. **g**, Final 3D map rendered at two contour levels and color-coded in a rainbow gradient according to radial direction. **h**, Six varied perspectives of the final map. **i**, The 3D map with an overlaid fitting model illustrating the structural model.

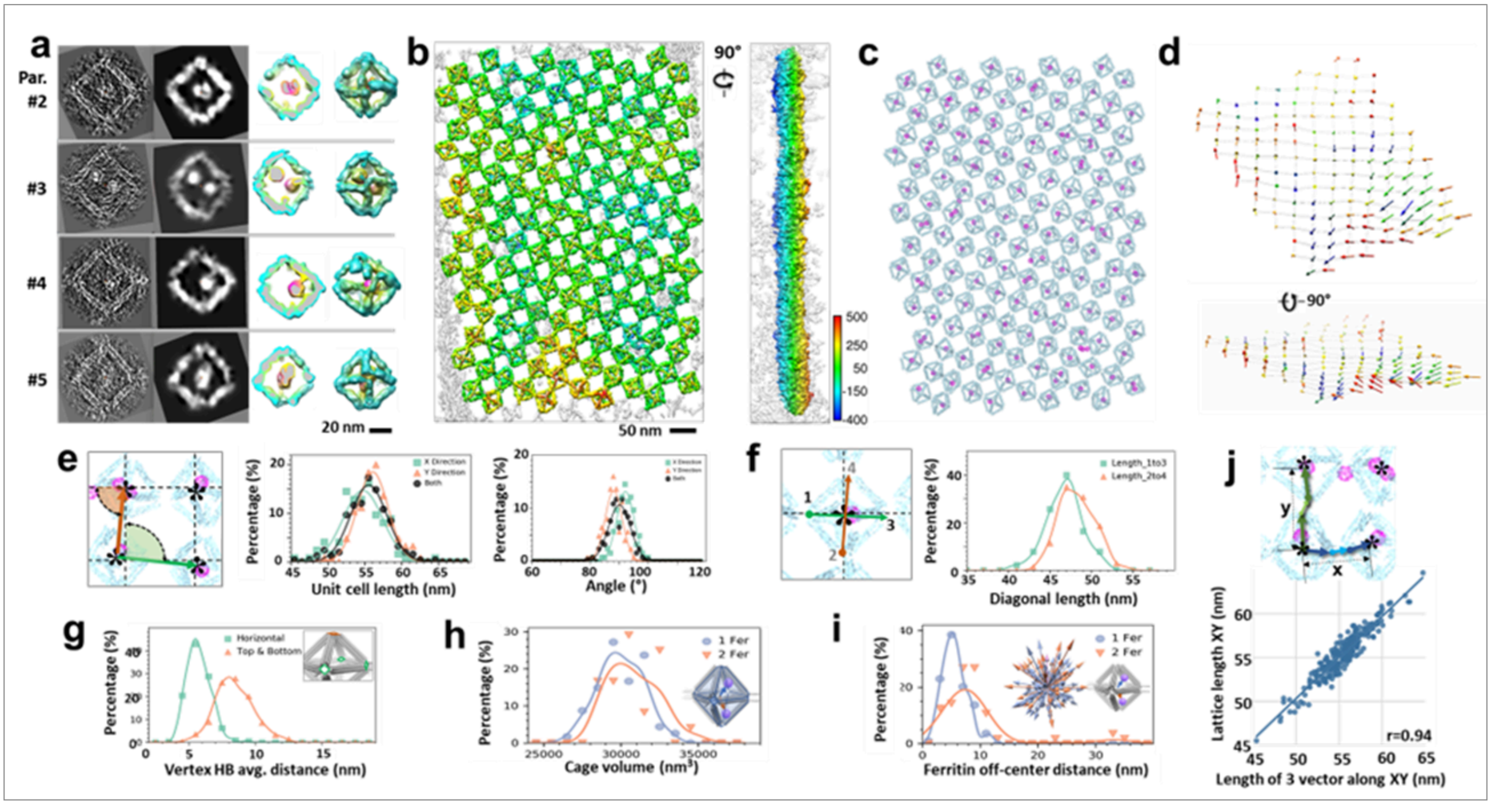

Figure 2

**3D structure and variability analysis of a 2D DNA origami lattice fully loaded with ferritin. a**, Four representative IPET reconstructions of unit-cell particles within a sample lattice (details in **Supp. Fig. 2**), shown as central slices of the 3D reconstruction before and after masking and low-pass filtering. There are also compared with the central 3D slices and full 3D views of the final maps. **b**, Two perpendicular views of the 3D reconstruction of an entire 2D lattice, restored from 140 IPET 3D maps reconstructed from its 140 unit-cell particles. Color-coded based on depth along the Z-axis. **c**, A fitting model of the 2D lattice, with ferritin particles represented in purple. **d**, Lattice disorder visualized by displacement vectors originating from the centers of individual unit-cells. The vectors point from the averaged lattice center, with displacements displayed as three times the positional offset vectors, represented in a rainbow color scale from negative (blue) to positive maxima (red). **e,** Histograms of unit-cell particle sizes, specifically vertex-to-vertex distances along the two diagonal directions within the quadrilateral formed by four HBs. **f**, Histograms of port sizes at the vertices, measured by averaging the distances among the four distal ends of the HBs at each vertex. **g**, Histograms of unit-cell particle volumes occupied by one or two ferritin particles. **h**, Histograms of ferritin displacement from the center of its unit-cell particle. **i**, Histograms of unit-cell lengths and angles measured between the centers of adjacent unit-cell particles along the X and Y axes. **j**, Correlation analysis between unit-cell particle center-to-center distances and the combined distances of vectors composed of two halves of adjacent unit-cell particle sizes plus their linker, measured along the X or Y axes, respectively.

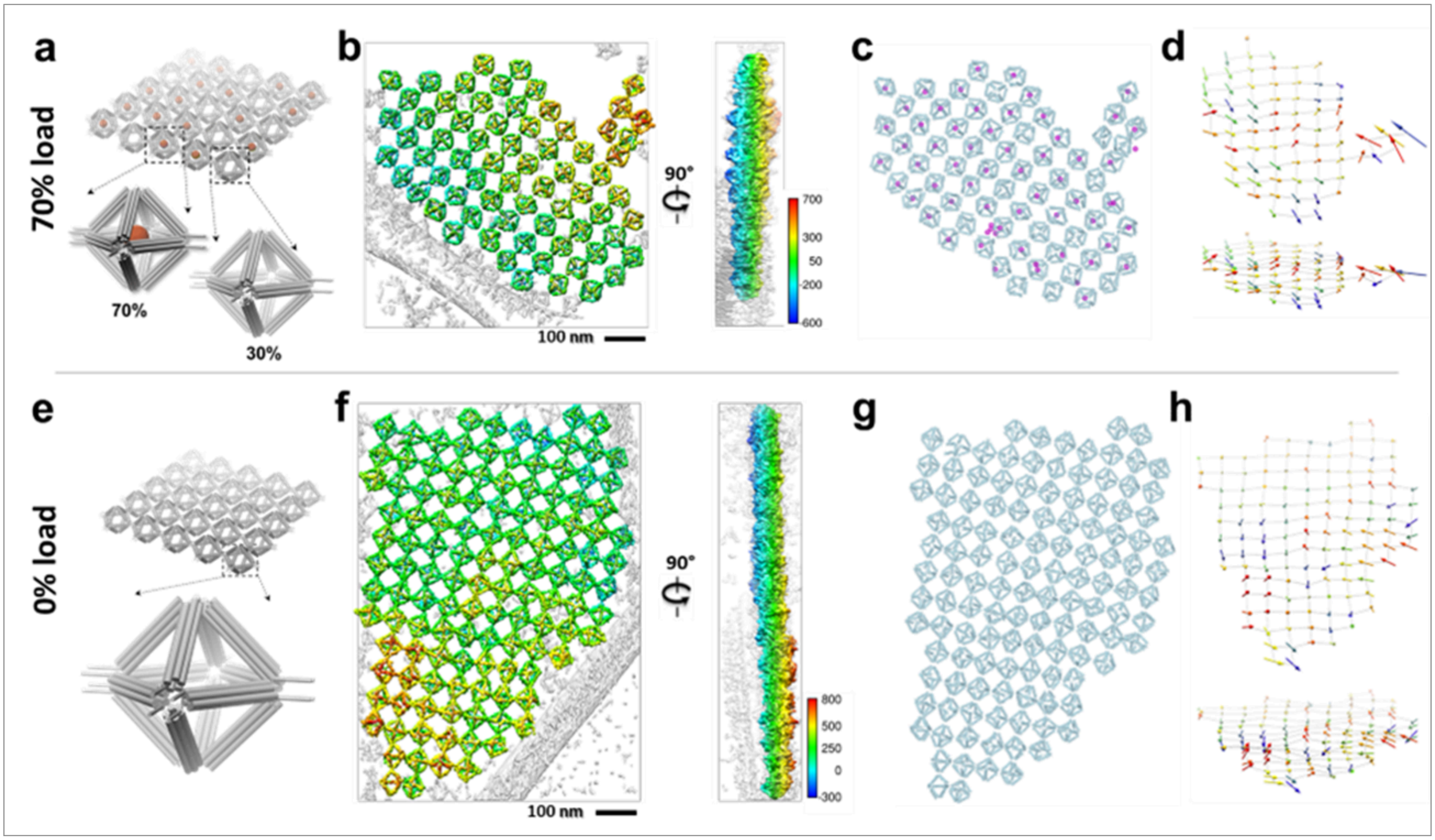

Figure 3

**3D structures of DNA origami 2D lattices with 70% and 0% ferritin loading. a**, Schematic representation of the DNA-origami 2D lattice with 70% ferritin loading. **b**, 3D reconstruction of a DNA-origami 2D lattice with 70% ferritin loading, displayed in perpendicular views. **c**, A fitting model of this lattice, with ferritin particles shown in purple. **d**, Lattice disorder visualized by displacement vectors originating from the centers of individual unit-cells. Color-coded arrows represent displacement vectors scaled to 3 times the positional offsets. **e-h**, Structure and model of a DNA-origami 2D lattice in the absence of ferritin loading.

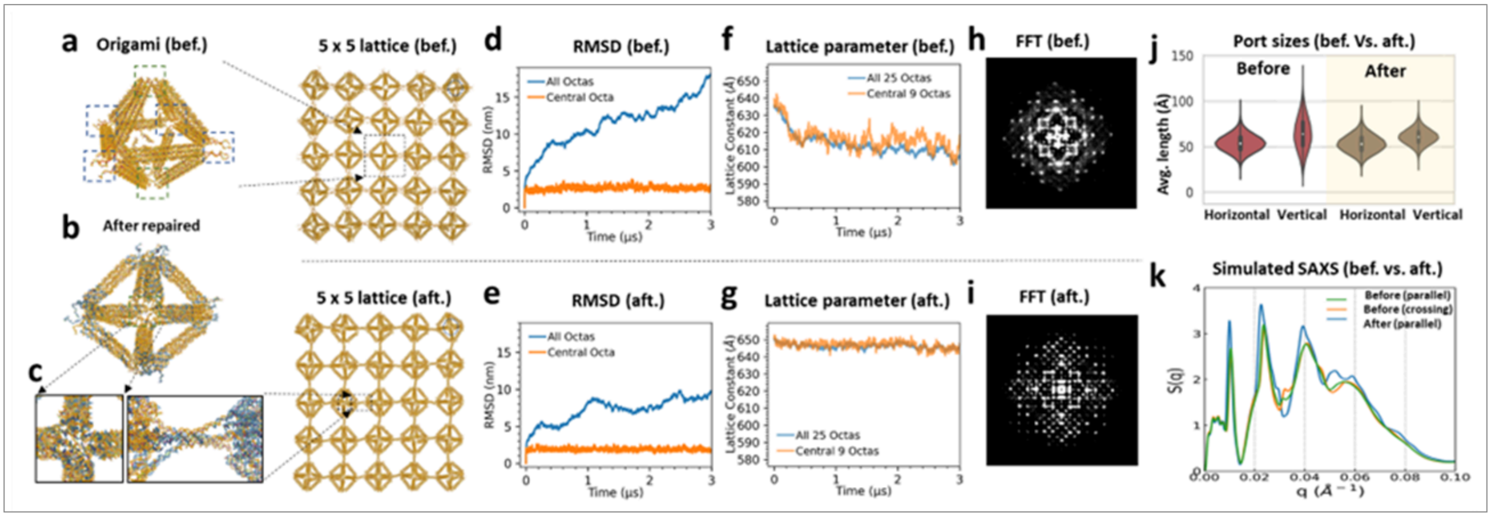

Figure 4

**Molecular dynamics (MD) simulations of DNA origami 2D lattices before and after repair. a**, Model of the DNA origami octahedral structure and it formed 2D lattice. **b**, Repaired DNA origami octahedral structure, achieved by introducing three additional DNA staples. **c**, The additional DNA staples included two staples added at the two off-plane vertices (left) and one staple added at the linkers (right), forming a repaired octahedral structure and its 2D lattice. **d**, Root Mean Square Deviation (RMSD) analysis of the original 2D lattice during a 3 μs MD simulation. **e**, RMSD analysis of the repaired 2D lattice during a 3 μs MD simulation. **f,g**, The analysis of unit-cell lengths in the lattices before and after repair during the 3 μs MD simulation. **h,i**, Fourier transform patterns of the 2D lattices before and after repair during MD simulations. **j** Distribution of pore sizes (average distances among the four distal ends of helical bundles at each vertex) before and after repair. **k**, Simulated Small-Angle X-ray Scattering (SAXS) curves of the lattices before and after repair, highlighting the structural improvements.

## Supplementary Files

This is a list of supplementary files associated with this preprint. Click to download.

- 01OctaFer100Low840.mp4
- 02OctaFer70Low840.mp4
- 03OctaOnlyLow840.mp4
- Lattice5x5EmptyMDBefore.avi
- DNAOrigami2DDynamicsSuppInfo20250223ver47.pdf
- ExtendedDataFigures.docx
